# Emergency curative resection of colorectal cancer, do it with caution. A comparative case series

**DOI:** 10.1016/j.amsu.2020.04.033

**Published:** 2020-05-13

**Authors:** Mohamed M. Elmessiry, Eman AE. Mohamed

**Affiliations:** a Department of Surgery, Alexandria Faculty of Medicine, Alexandria, Egypt; b Department of Internal Medicine, Alexandria Faculty of Medicine, Alexandria, Egypt

**Keywords:** Complicated colorectal cancer, Emergency curative resection, Surgical and oncologic outcomes

## Abstract

**Introduction:**

The feasibility and efficacy of emergency curative resection of complicated colorectal cancer is still controversial. This prospective study aim was to assess surgical and oncologic outcomes after emergency compared to elective curative resection of colorectal cancer.

**Methods:**

60 consecutive patients presented with complicated colorectal cancer managed by emergency surgery were included and compared to another 155 consecutive patients admitted during the same period with uncomplicated colorectal cancer managed by elective surgery. Both groups were compared regarding curative resection rate, early postoperative mortality and morbidity, 3-years tumor recurrence and survival rates.

**Results:**

Complicated colorectal cancer presented at a more advanced stage with a lower resectability rate and higher postoperative mortality and morbidity rates when compared to uncomplicated ones. Emergency resection of stage I/II colorectal cancer had similar 3-years disease free, overall survival and cancer-specific mortality rates approximating elective. But, emergency resection of stage III tumors had significantly decreased 3-years disease free and overall survival rates although there was no significant increase in cancer specific mortality rate.

**Conclusions:**

Complicated colonic cancers present at a more advanced stage with a lower resectability rate, and higher postoperative morbidity and mortality rates when compared with uncomplicated ones. In medically fit patients, emergency curative resection of complicated colorectal cancer could be done safely with survival outcomes approximating elective resection of uncomplicated cancer in the same stage if proper oncologic resection done by expert surgeon.

## Highlights

• The feasibility and efficacy of emergency curative resection of complicated colorectal cancer is still controversial.
• Most of previous studies were not well designed and included both curative and palliative resection.
• This study compared emergency and elective curative resection regarding proper resection, tumor recurrence & survival rate.
• This study revealed that emergency curative resection has ‎higher postoperative morbidity and mortality rates.
• Proper emergency resection by expert surgeon has similar survival outcomes to elective resection of cancer in same stage.

## 1 Introduction

Approximately 30% of patients with colorectal cancer present as an emergency by complications that may be the first presentation, with diagnosis, staging and assessment of resectability done intra-operatively [[1], [2], [3]].Urgent colorectal operations are associated with higher mortality and morbidity rates than elective surgery [[4], [5], [6]]. Many studies state that emergency colorectal resection is inadequate with poor oncologic outcomes [1,7] while, other studies claim that emergency curative resection of colorectal cancer has survival outcomes similar to elective resection [8].

**The aim** of this prospective study was to assess feasibility, surgical and oncologic outcomes after emergency compared to elective curative resection of colorectal cancer. The secondary aim was to identify predictors of poor outcomes after emergency resection to help in patient counseling and proper selection of candidate for emergency curative resection.

**Patients:** after approval by the Ethics Committee of Alexandria Faculty of Medicine, this study included 60 consecutive patients presented by complicated colorectal cancer treated by emergency surgery at Alexandria University hospital between January 2015 and January 2017 (Group I). Another 155 patients with uncomplicated colorectal cancer treated by elective surgery during the same period were included (Group II). During surgical exploration, 13 patients in emergency group and 9 patients in elective group were found to be inoperable and treated by palliative surgery and excluded. Another 2 patients in emergency group and 3 patients in elective group didn't complete the follow up and were excluded.

## 2 Methods

**Preoperative evaluation** of all patients included complete clinical examination, routine laboratory investigations and assessment of comorbidities. Plain abdominal X-ray in erect position was ordered if intestinal obstruction was suspected. CT abdomen and pelvis, colonoscopy and histopathologic assessment of colonoscopic biopsy, and MRI for accurate staging of rectal cancer were done whenever possible. Preoperative management included fluid resuscitation, colonic preparation when possible, antibiotic and thrombo-prophylaxis. All patients signed a preoperative consent including the possibility of intestinal stoma.

The surgical procedures were determined by tumor location, intraoperative staging and assessment of resectability, associated medical diseases and risk of the procedure. Tumors were considered incurable if there were multiple peritoneal deposits, multiple liver metastasis or irresectable locally advanced tumor invading critical adjacent structures not amenable for radical resection. Right or extended right hemicolectomy was done for resectable right sided tumors, while, ileocolic bypass was done for irresectable ones. For resectable left sided tumor, resection and primary anastomosis was performed in fit patients with unloaded colon without risk of anastomotic leak while, Hartmann's procedure was performed in unfit patients with loaded colon or high risk of leak. Intraoperative colonic lavage was done before primary anastomosis after resection of left sided tumors in fit patients with mildly loaded colon. Total colectomy was done in fit patients with heavily loaded colon, impending perforation of the dilated cecum, or suspicious multiple synchronous tumors. Temporary intestinal stoma was performed after curative resection anastomosis for resectable left sided tumors with moderate risk of anastomotic leak. Permanent stoma was performed for irresectable tumors or after curative abdominoperineal resection. Both groups were compared regarding: resectability, early postoperative mortality and morbidity rates. Surgical specimens in both groups were examined and compared regarding proper oncologic resection including adequate distal and circumferential resection margins, integrity of mesocolon or mesorectum, and adequate lymphadenectomy (number of LN harvests more than 12 LNs). Moreover, tumor characteristics in both groups were compared regarding tumor size, grade and stage, lymphatic or venous invasion, nodal stage and TNM stage.

Follow up was done every 3 months during the first year then every 6 months after that. The follow up included clinical assessment and abdominal CT if needed. After 3 years follow up, both groups were compared regarding tumor recurrence, disease free survival (DFS) and overall survival (OS) rates in correlation with clinicopathological characteristics.

### 2.1 Statistical analysis

Numerical data in both groups was expressed as mean ± standard deviation (SD) and compared using One-way analysis of variance while categorical data was expressed as percentages and compared using Chi-squared test. Survival outcomes were compared using Kaplan Meier method. Multivariate analysis was used to identify predictors of poor outcomes. Differences were considered significant at p < 0.05.

## 3 Results

The demographic and clinical characteristics of patients in both groups were matched except that patients’ age in emergency group was significantly older (p 0.001). The main presenting symptom in emergency group was intestinal obstruction (75%) followed by colonic perforation (16.7%) while in elective group it was rectal bleeding (23.2%) followed by anemia with occult blood in stool (20.6%). The laboratory workup showed significant leukocytosis and hypoaluminamia in emergency group. Urgent colonoscopy was done in 5 patients (8.33%); while, all patients in elective group had colonoscopy done with accurate diagnosis and staging before surgery. All patients in elective group had colonic preparation before surgery compared to only 4 patients in emergency group (Table 1).

**Table 1:** Preoperative patients’ characteristics in both groups.

		Elective (n = 155)		Emergency (n = 60)		X^2^/F	p
		N	%	N	%		
Gender	Male	82	52.9%	36	60.0%	0.88	0.348
	Female	73	47.1%	24	40.0%		
Age (mean ± SD)		57.52 ± 8.916		64.09 ± 10.74		16.801	0.001*
BMI (mean ± SD)		27.48 ± 5.458		26.75 ± 6.743		0.543	0.462
ASA score	ASA 1 A normal healthy patient.	51	32.9%	12	20.0%	4.003	0.135
	ASA 2 A patient with mild comorbidity	71	45.8%	30	50.0%		
	ASA 3 A patient with severe comorbidity	33	21.3%	18	30.0%		
Presenting Symptoms	Intestinal obstruction	0	0.0%	45	75.0%	205.582	0.001*
	Rectal bleeding	36	23.2%	2	3.3%		
	Colonic perforation and peritonitis	0	0.0%	10	16.7%		
	Acute appendicitis	0	0.0%	3	5.0%		
	Anemia + occult blood in stool	32	20.6%	0	0.0%		
	Weight loss – weakness	18	11.6%	0	0.0%		
	Abdominal mass	29	18.7%	0	0.0%		
	Asymptomatic(accidental during imaging)	40	25.8%	0	0.0%		
Preoperative colonoscopy & biopsy	Not available	0	0.0%	55	91.7%	190.924	0.001*
	Available	155	100.0%	5	8.3%		
Preoperative Hb (mean ± SD)		10.46 ± 0.911		10.08 ± 0.909		3.397	0.067
Preoperative WBC (mean ± SD)		8.71 ± 1.563		11.50 ± 1.830		106.465	0.001*
Preoperative Albumin (mean ± SD)		3.09 ± 0.347		2.95 ± 0.204		7.482	0.007*
Preoperative Bowel Preparation	No	0	0.0%	56	93.3%	195.61	0.001*
	Yes	155	100.0%	4	6.7%		

**Surgical outcomes:** (Table 2): 47/60 patients (78.3%) in emergency group had a potentially curative resection compared to 146/155 patients (94.2%) in elective group (P 0.001). Palliative resection was done in 5 patients (8.3%) in the emergency compared to 3 patients (1.9%) in elective group. The rate of palliative stoma and stoma after curative resection were 6.7% and 36.7% in emergency group compared to 3.2% and 18.1% in elective group, p 0.047. The mean operative time was significantly higher during emergency curative resection (168.11 ± 28.74 versus 145.03 ± 26.45, P 0.001). Also, operative bleeding was slightly increased during emergency curative resections (188.00 ± 100.38 versus 163.43 ± 74.41, P 0.079). **Early postoperative mortality** after curative colorectal resection was more frequent in emergency compared to elective group with no statistically significant difference, 11.1% versus 4.9%, P 0.137. The cause of early postoperative mortality was related to surgical complications (sepsis) in 3 patients in each group while it was related to medical comorbidities in 2 patients in emergency group and 4 patients in elective group with no statistically significant difference (P 0.561). **Early postoperative complications** after curative colorectal resection were more frequent in emergency compared to elective group with no statistically significant difference, 26.7% versus 15.4%, P 0.086. The majority of early postoperative complications after curative colorectal resection in both groups was associated with old age (p < 0.001), high BMI (p < 0.001), higher ASA score (p < 0.001), hypoalbuminamia (p 0.047), tumors presented with perforation and peritonitis (p 0.042), advanced tumor stage (p 0.033). The mean length of hospital stay was more in the emergency compared to elective group with no statistically significant difference, (7.18 ± 5.49 versus 6.24 ± 2.78, P 0.13).

**Table 2:** Operative data in both groups.

		Elective (n = 155)		Emergency (n = 60)		X^2^/F	P
		N	%	N	%		
1ry Tumor Site	Rectum	53	34.2%	8	13.3%	15.722	0.015*
	Sigmoid	34	21.9%	21	35.0%		
	Left colon	24	15.5%	8	13.3%		
	Splenic flexure	5	3.2%	7	11.7%		
	Transverse colon	5	3.2%	3	5.0%		
	Hepatic flexure	6	3.9%	3	5.0%		
	Right colon	28	18.1%	10	16.7%		
1ry Tumor Side	Left	116	74.8%	44	73.3%	0.095	0.757
	Right	39	25.2%	16	26.7%		
Peritonitis	No	155	100.0%	53	88.3%	18.692	0.001*
	Yes	0	0.0%	7	11.7%		
Fixation to adjacent structure amenable for resection	No	144	92.9%	49	81.7%	9.094	0.105
	Abdominal/pelvic wall	3	1.9%	3	5.0%		
	UB	3	1.9%	2	3.3%		
	Uterus/Vagina	2	1.3%	1	1.7%		
	SI	3	1.9%	3	5.0%		
	Spleen	0	0.0%	2	3.3%		
Fixation to adjacent structure not amenable for resection	No	152	98.1%	54	90.0%	7.428	0.059
	Major vessels - Posterior Abdominal/pelvic wall	1	0.6%	2	3.3%		
	Ureter - Kidney	1	0.6%	3	5.0%		
	Pancreas –Dudenum	1	0.6%	1	1.7%		
Peritoneal deposits	No	146	94.2%	48	80.0%	13.083	0.001*
	Localised	0	0.0%	3	5.0%		
	Generalised	9	5.8%	9	15.0%		
Liver metastases	No	152	98.1%	50	83.3%	16.81	0.001*
	Solitary	0	0.0%	1	1.7%		
	Multiple	3	1.9%	9	15.0%		
Surgery intent	Palliative stoma/bypass without resection	6	3.9%	8	13.3%	17.031	0.001*
	Palliative resection	3	1.9%	5	8.3%		
	Curative resection	146	94.2%	47	78.3%		
Surgical procedure	No resection - Diverting stoma/bypass	6	3.9%	8	13.3%	51.220	0.001*
	Rt/Extended Rt hemicolectomy - Ileocolic Anastomosis	41	26.4%	16	26.7%		
	Lt colectomy/Sigmoidectomy/AR/LAR of rectum - Colostomy	5	3.2%	17	28.4%		
	Lt colectomy/Sigmoidectomy/AR/LAR of rectum – 1ry Anastomosis	72	46.5%	12	20%		
	Lt colectomy/Sigmoidectomy/AR/LAR of rectum – 1ry Anastomosis + Covering stoma	22	14.2%	3	5.0%		
	APR of rectum - Colostomy	6	3.9%	2	3.3%		
	Total colectomy - Ileorectal Anastomosis	3	1.9%	2	3.3%		
Intraoperative on table lavage	No	155	100.0%5	47	78.3%	35.745	0.001*
	Yes	0	0.0%	13	21.7%		
Stoma	None	122	78.7%	34	56.6%	5.171	0.047*
	Palliative stoma without resection	5	3.2%	4	6.7%		
	Stoma after curative resection	28	18.1%	22	36.7%		

**Histopathologic assessment of surgical specimens (**Table 3**)** revealed that patients with emergency resection had significantly larger tumor size, higher tumor grade and advanced both tumor and nodal stage. Regarding proper oncologic resection; there was a tendency for circumferential resection margin infiltration in emergency group (P 0.055). Number of LN harvest was statistically less in emergency when compared to elective group (11.35 ± 2.25 versus 15.88 ± 2.68, P < 0.001).

**Table 3:** Pathological data of surgical specimens after curative resection in both groups.

		Elective (n = 143)		Emergency (n = 45)		X^2^/F	p.
		N	%	N	%		
T Size (mean ± SD)		3.39 ± 0.712		4.59 ± 0.881		86.400	0.001*
T Grade	Well differentiated	84	58.7%_a_	11	24.4%_b_	21.43	0.001*
	Moderately differentiated	45	31.5%_a_	19	42.2%_a_		
	Poorly differentiated	14	9.8%_a_	15	33.3%_b_		
T Stage	T2	63	44.1%	1	2.2%	40.664	0.001*
	T3	71	49.7%	28	62.2%		
	T4	9	6.3%	16	35.6%		
Lymphatic/Vascular Invasion	No	106	74.1%_a_	24	53.3%	6.937	0.008*
	Yes	37	25.9%_a_	21	46.7%		
Circumferential margin	Free	141	98.6%_a_	42	93.3%_a_	3.669	0.055
	Infiltrated	2	1.4%_a_	3	6.7%_a_		
Mesorectal/Mesocolic Excision	Incomplete	9	6.3%_a_	7	15.6%_a_	3.771	0.052
	Complete	134	93.7%_a_	38	84.4%_a_		
No LN Harvest	<12 LN	12	8.4%	23	51.1%	41.229	0.001*
	>12 LN	131	91.6%	22	48.9%		
No LN Harvest (mean ± SD)		15.88 ± 2.6898		11.35 ± 2.2547		103.490	0.001*
pN stage	N0	104	72.7%_a_	22	48.9%	8.800	0.003*
	N1/2	39	27.3%_a_	23	51.1%		
TNM stage	Stage I – II	105	73.4%	20	44.4%	24.162	0.001*
	Stage III	38	26.6%	25	55.6%		

**Oncologic outcomes** (Table 4a, Table 4b, d):45 patients treated by emergency curative resection had completed the follow up for about 3 years, 25 patients 55.6% were staged as stage III and 20 patients (44.4%) a stage I/II, while in elective group, 143 had complete follow up, 38 patients (26.6%) in stage III and 105 patients (73.4%) in stage I/II. 3-years recurrence rate was significantly higher after emergency curative resection (28.9%% versus 8.4%, P 0.001). Using Multivariate analysis, predictors of recurrence included high tumor grade (p 0.045), presence of lymphatic/venous invasion (p 0.012), advanced tumor stage (p 0.031), advanced nodal stage (p 0.002), infiltrated resection margins (p < 0.001), inadequate lymphadenectomy with LN retrieved < 12 (p 0.001), and incompleteness of mesorectum (p 0.001). 3-years DFS was significantly decreased following emergency compared to elective curative resection, 64.4% versus 87.4%, P 0.001. 3-years Cancer specific mortality was significantly increased after emergency resection, 15.6% versus 5.6%, p0.032. Also, 3-years OS rate was significantly decreased following emergency resection, 71.1% versus 88.8%, P 0.016. **In patients with stage I/II** colorectal cancer, there was no significant difference in oncologic outcomes after emergency compared to elective curative resection. On the other hand**, in patients with stage III tumors,** 3-years DFS rate was significantly decreased after emergency surgery compared to elective surgery (56% versus 86.8, P 0.006). Also, 3-years OS rate was significantly decreased after emergency surgery (60% versus 86.8%, P 0.015). The majority of overall mortality was associated with old age, higher ASA score (P 0.040), advanced tumor stage (P < 0.001) and tumor recurrence (P 0.001).

**Table 4a:** Oncologic outcomes after curative colorectal resection in both groups.

		Elective (n = 143)		Emergency (n = 45)		X^2^/F	P
3 y Tumor recurrence	No	131	91.6%	32	71.1%	12.473	0.001*
	Yes	12	8.4%	13	28.9%		
3 y DFS	No	18	12.6%	16	35.6%	12.189	0.001*
	Yes	125	87.4%	29	64.4%		
3 y cancer specific Mortality	No	135	94.4%	38	84.44%	6.525	0.032*
	Yes	8	5.6%	7	15.56%		
3 y OS	No mortality	127	88.8%	32	71.1%	8.278	0.016*
	Died from cancer	8	5.6%	7	15.6%		
	Died from other cause	8	5.6%	6	13.3%

**Table 4b:** Oncologic outcomes after curative colorectal resection in both groups in correlation with TNM stage.

		Curative resection in stage I and II						Curative resection in stage III					
		Elective (n = 105)		Emergency (n = 20)		X^2^/F	P	Elective (n = 38)		Emergency (n = 25)		X^2^/F	P
3 y Tumor recurrence	No	99	94.3%	16	80.0%	4.658	0.031	32	84.2%	16	64.0%	3.395	0.065
	Yes	6	5.7%	4	20.0%			6	15.8%	9	36.0%		
3 y DFS	No(rec or death)	13	12.4%	5	25.0%	2.170	0.141	5	13.2%	11	44.0%	7.571	0.006*
	Yes	92	87.6%	15	75.0%			33	86.8%	14	56.0%		
3 y cancer specific mortality	No	101	96.2%	19	95.0%	0.062	0.803	34	89.5%	18	72.0%	3.195	0.074
	Yes	4	3.8%	1	5.0%			4	10.5%	7	28.0%		
3 y OS	No mortality	94	89.5%	17	85.0%	0.133	0.935	33	86.8%	15	60.0%	8.424	0.015*
	Died from cancer	4	3.8%	1	5.0%			4	10.5%	6	24.0%		
	Died from other cause	7	6.7%	2	10.0%			1	2.6%	4	16.0%

**Fig. 1:**
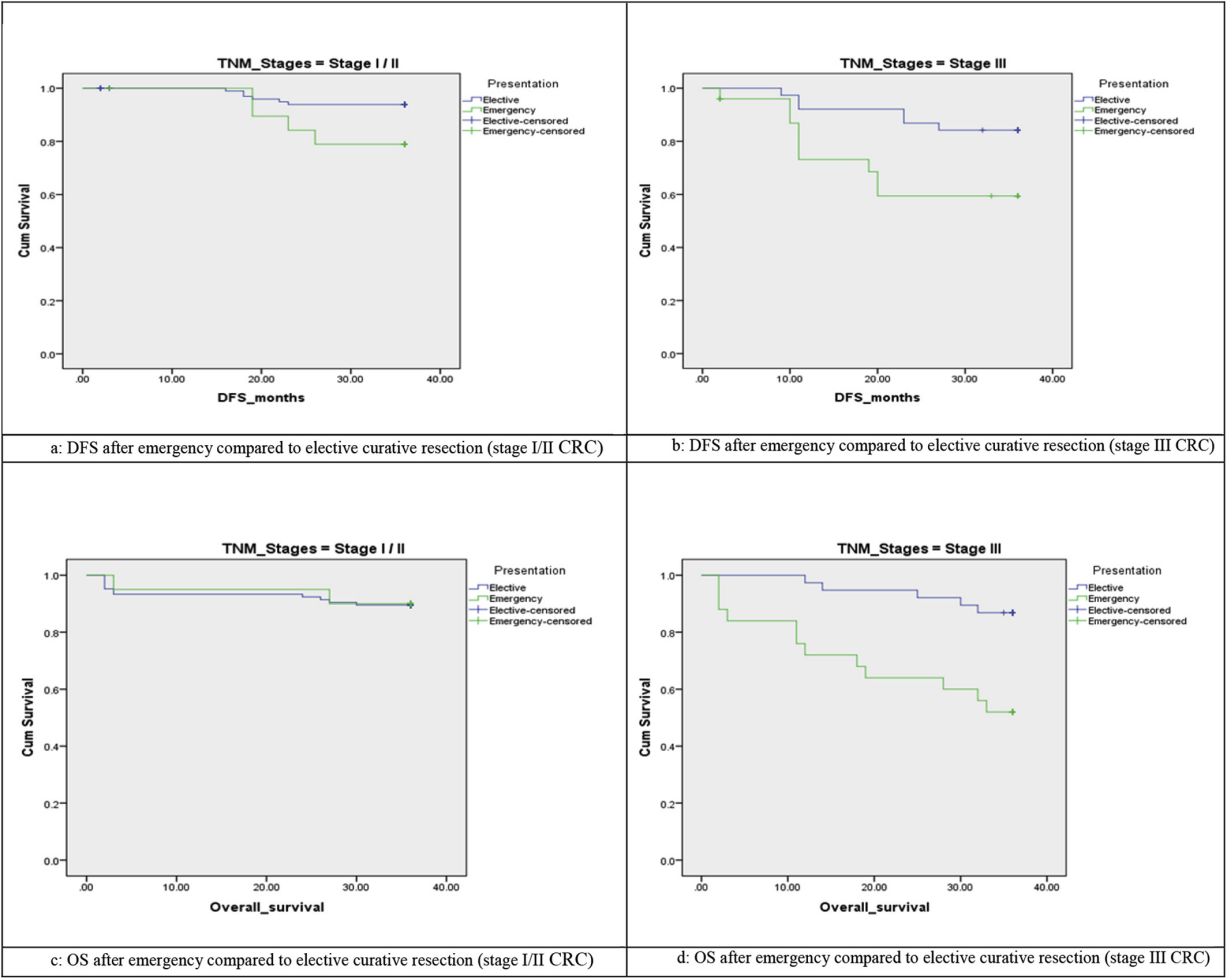
Survival curves after curative resection of colorectal cancer in the same TNM stage in both groups Fig. 1a: DFS after emergency compared to elective curative resection of stage I/II tumors. Fig. 1b: DFS after emergency compared to elective curative resection of stage III tumors. Fig. 1c: OS after emergency compared to elective curative resection of stage I/II tumors. Fig. 1d: OS after emergency compared to elective curative resection of stage III tumors.

## 4 Discussion

The feasibility and outcomes after emergency curative resection of complicated colorectal cancer is still debatable. This study was done to compare surgical and oncologic outcomes after emergency versus elective curative resection of colorectal cancer.

In our study groups, the demographic and clinical characteristics of patients in both groups were nearly matched but patients in emergency group were older with tumors located mainly in sigmoid (35%) while rectal cancer was less in emergency compared to elective group (13.3% versus 34.2%), in agreement with the finding of previous studies that complicated colorectal cancer are more common with old age and less frequent in the rectum [9,10]. The main emergency clinical presentation in our study was intestinal obstruction (75%) followed by perforation (16.7%) while, in elective group, main presenting symptoms were rectal bleeding (23.2%) or anemia with occult blood in stool (20.6%). Wong et al. [10] found that the main reason for emergency surgery was bowel obstruction (78%) then perforation (10%)

In our study, curative resection was feasible in 78.3% of patients in emergency group compared 94.2% in elective group while palliative resection was done in 8.3 compared to 1.9%. The rate of palliative stoma and stoma after curative resection were 6.7% and 36.7% in emergency group compared to 3.2% and 18.1% in elective group. Similarly, Pavlidis et al. [9] reported a lower resectability rate in emergency compared to elective surgery of colorectal cancer, (75% versus 90%). Also, the rate of palliative stoma and stoma after curative resection in Pavlidis study were 19% and 28% in emergency group compared to 2.7% and 6% in elective group [9]. The higher stoma rate in our study is explained by our trend to perform protective stoma in most of the cases treated by AR/LAR especially in the emergency setting with hypoalbuminamia. In our study, the mean operative time and mean intraoperative blood loss were significantly higher during emergency compared to elective resection. This may be explained by the high percentage of advanced tumor (stage III) in emergency compared to elective group.

In the current study, early postoperative mortality and early postoperative complications were higher after emergency compared to elective group but with no statistically significant difference and the length of hospital stay was significantly increased only in patients developed postoperative complications. Biondo et al. [11], reported significantly higher early postoperative mortality rate after emergency compared to elective colorectal resection 15.3*%* versus 4.8*%.* Many other studies confirmed that emergency colorectal resection is associated with higher postoperative morbidity rate and longer hospital stay especially in left sided tumor [3,5,6,12]. In our study, the majority of early postoperative complications after curative colorectal resection in both groups were associated with old age, high BMI, higher ASA score, hypoalbuminamia, tumors presented with perforation and peritonitis and advanced tumor stage. Similar results was reported by Biondo et at, who concluded that age, advanced malignant disease, physiologic status, and systemic sepsis contribute to the high morbidity and mortality rates [13]. Verbo et al. used colorectal tumors emergency score (CTES), based on 4 risk factors, namely colonic perforation, serum albumin, concurrent cardiovascular disease and chronic renal insufficiency. Patient is ranked low (CTES <4), moderate (CTES 4–12) or high (CTES >12). High risk patients are better to be treated by a staged procedure, moderate risk patients may be treated by immediate resection of the tumor, without anastomosis, while low risk patients could be treated by immediate resection and anastomosis [14].

Histopathologic assessment of surgical specimens revealed that patients with emergency presentation had significantly larger tumor size, higher grade and more advanced pathological tumor and nodal stage. Similar results were documented in many other studies [11,12,15]. It seems reasonable that locally advanced tumors, by infiltrating through the bowel wall, could result in obstruction or perforation. A locally advanced tumor would also be more likely to display venous or lymphatic invasion, which increase the probability of lymph node metastases, as indicated by the N stage.

Regarding proper oncologic resection there was a tendency for circumferential resection margin infiltration after emergency compared to elective resection. Similar finding was reported by Ghazi et al. [15]. Inadequate lymphadenectomy “LN harvest <12 LN” was more frequent in emergency compared to elective resection in our study, although previous studies found no difference in the mean number of LN harvest between emergency and elective colorectal resection [10,12].

In the present study, there was significantly increase in 3-years recurrence rate and decrease in 3-years DFS and OS rates after emergency compared to elective curative resection **w**hich could be explained by the higher percentage of patients with stage III in emergency group. This agrees with previous studies which concluded that emergency colorectal resection may be inadequate with higher recurrence rates and poor survival outcomes. [7,8,16]. Using multivariate analysis, predictors of recurrence included high tumor grade, presence of lymphatic/venous invasion, advanced pathological tumor and nodal stages, positive resection margins, and incompleteness of mesorectum. Similar data was reported by Cortet et al., who stated that age and tumor stage were predictors of local recurrence after curative resection of obstructed colorectal cancer [16]. Merkel et al. [3] found that high recurrence rates after emergency colorectal surgery was associated with tumor stage and infiltrated resection margins. In agreement with literature data [10], the majority of overall mortality in our study was associated with old age, higher ASA score, advanced Tumor stage, tumor recurrence.

When comparing patients with the same TNM stage we found no significant difference in oncologic outcomes after emergency compared to elective surgery in patients with stage I or II tumors, there agrees with other studies which concluded that emergency curative resection of colorectal cancer has similar survival outcomes to those treated by elective resection with the same tumor stage [8,17]. But, patients with stage III tumors in our study had significantly decreased 3-years DFS and OS rates when compared to elective resection. This difference could be explained by the fact that most of the elective resections were done by more expert surgeons compared to emergency group which was reflected on better oncologic resection in terms of adequate lymphadenectomy, circumferential margins and complete mesorectal excision.

### 4.1 Limitations of the study

First, there is a risk of selection bias; most of patients admitted for elective curative resection had better ASA score compared to emergency group because they had time for preoperative management of their comorbidities. Secondly, there is a risk of variability in surgeons experience; most of the elective resections were done by more expert surgeons compared to emergency group which was reflected on better oncologic resection in terms of adequate lymphadenectomy, circumferential margins and complete mesorectal excision. Lastly, there is a risk of inter-observer variability; the surgical specimens were assessed by different pathologist with different experience that could be reflected on the evaluation of the proper oncologic resection and tumor characteristics.

## 5 Conclusion

Complicated colonic cancers present at a more advanced stage with a lower resectability rate, and higher postoperative mortality and morbidity rates when compared with uncomplicated ones. In medically fit patients, emergency curative resection of complicated colorectal cancer could be done safely by expert surgeon with survival outcomes approximating elective resection of uncomplicated cancer in the same stage.

## 6 Recommendation

A larger study is recommended with a good quality control including all emergency and elective resections done by expert colorectal surgeons with proper oncologic resection in terms of adequate lymphadenectomy, circumferential margins and complete mesorectal excision. Also, the surgical specimens assessed by a single expert pathologist to avoid the risk of inter-observer variability.

## 7 Statement of ethics

The Research Ethics Committee of Alexandria Faculty of Medicine has reviewed and approved this study. Informed written consent was taken from all patients before being included in this study. The study is registered in ClinicalTrials.gov with Id: NCT04288284. This work has been reported in line with the PROCESS 2018 criteria [18].

## Funding source

None.

## Provenance and peer review

Not commissioned, externally peer reviewed.

## Declaration of competing interest

The authors declare that they have no conflicting interests.
